# Potent and Sustained Lowering of Serum Uric Acid by YJH‐012‐D, a GalNAc‐Conjugated siRNA Targeting Xanthine Dehydrogenase

**DOI:** 10.1002/prp2.70308

**Published:** 2026-07-31

**Authors:** Tingke Tang, Qiuying Li, Chenguang Zhang, Yongxing Wang, Lu Yang, Haibin Huang, Yujun He, Zhi Zheng, Dong Liang, Meng Wang, Yingyu Wu, Chengjiang Zhao, Wenhao Cui

**Affiliations:** ^1^ School of International Pharmaceutical Business, China Pharmaceutical University Nanjing Jiangsu China; ^2^ Youjia (Hangzhou) Biomedical Technology Co. Ltd Hangzhou Zhejiang China; ^3^ Livzon Pharmaceutical Group Inc Zhuhai Guangdong China; ^4^ Jiangxi Provincial People's Hospital Nanchang Jiangxi China; ^5^ General Practice Department Tianjin First Central Hospital Tianjin China

**Keywords:** hyperuricemia, siRNA therapeutics, urate‐lowering therapy, xanthine dehydrogenase

## Abstract

Hyperuricemia (HUA) is primarily driven by hepatic overproduction of urate, mediated by xanthine dehydrogenase (XDH). This preclinical study evaluates YJH‐012‐D, a *N*‐acetylgalactosamine (GalNAc)‐conjugated small interfering RNA (siRNA) targeting hepatic *XDH*. Functional assays demonstrated that YJH‐012‐D potently suppressed both *XDH* mRNA and protein expression in primary hepatocytes. In mice, a single subcutaneous dose of 8 mg/kg significantly reduced serum uric acid levels and suppressed hepatic XDH expression for more than 42 days. In cynomolgus monkeys, administration of YJH‐012‐D at 10 mg/kg lowered serum uric acid for up to 180 days, with sustained XDH inhibition observed for 120 days. Pharmacokinetic analysis revealed a high liver‐to‐plasma area under the curve (AUC) ratio (2000–6100) and a hepatic half‐life exceeding 10 days, consistent with prolonged pharmacological activity. Comprehensive toxicity assessments—including clinical biochemistry, histopathology, and RNA sequencing (RNA‐seq)‐based off‐target profiling—identified no significant adverse effects. Collectively, YJH‐012‐D exhibits potent, durable urate‐lowering efficacy and a favorable safety profile, underscoring its potential as a novel therapeutic candidate for HUA.

## Graphical abstract

We developed a GalNAc‐conjugated siRNA targeting hepatic XDH, which effectively reduces uric acid production by silencing XDH expression, offering a promising therapeutic approach for hyperuricemia.

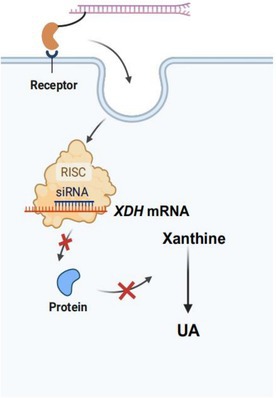

## 1 Introduction

Hyperuricemia (HUA) is a chronic metabolic disorder characterized by abnormally elevated serum uric acid levels. Its global prevalence is rising, particularly in China, in parallel with population aging and changes in lifestyle [1]. A nationwide survey spanning 13 provinces reported a prevalence of 6.4% among Chinese adults [2]. Although many individuals erroneously perceive asymptomatic hyperuricemia as benign, accumulating evidence indicates that HUA is closely associated with metabolic syndrome, type 2 diabetes, hypertension, cardiovascular disease, chronic kidney disease, and gout [3, 4].

Uric acid, the terminal product of purine metabolism in humans, is generated through both exogenous dietary intake and endogenous biosynthesis of purines. Xanthine oxidoreductase (XOR)—which exists in two interconvertible forms, xanthine dehydrogenase (XDH) and xanthine oxidase (XO)—catalyzes the final steps of purine catabolism and thus plays a pivotal role in uric acid production [5, 6]. Clinically, urate‐lowering therapy primarily relies on xanthine oxidase inhibitors, such as allopurinol and febuxostat [7]. However, these agents are frequently limited by serious adverse effects, including allopurinol hypersensitivity syndrome (AHS) [8] and increased cardiovascular risk with febuxostat [9], underscoring the urgent need for safer and more effective therapeutic strategies. Notably, hepatic‐specific knockout of XDH in mice has been shown to markedly reduce uric acid concentrations in both blood and liver.

In recent years, small interfering RNA (siRNA) therapeutics have emerged as a powerful platform for precision gene silencing, offering novel avenues for treating a wide range of diseases [10]. siRNA mediates sequence‐specific degradation of target mRNA, thereby suppressing the expression of pathogenic proteins at the transcriptional level. Notably, hepatic‐specific knockout of XDH in mice has been shown to markedly reduce uric acid concentrations in both blood and liver [11], suggesting that siRNA‐mediated inhibition of XDH could represent a rational approach to treating HUA by targeting uric acid synthesis at its primary source—potentially circumventing the off‐target toxicities of conventional small‐molecule inhibitors. To enable efficient in vivo delivery, N‐acetylgalactosamine (GalNAc) conjugation has become a leading strategy for hepatocyte‐directed nucleic acid therapeutics [12]. GalNAc ligands bind with high affinity to the asialoglycoprotein receptor (ASGPR), which is abundantly and selectively expressed on hepatocytes, facilitating robust and liver‐specific uptake of siRNA payloads [13, 14]. To date, seven GalNAc‐conjugated siRNA drugs have received regulatory approval, with numerous candidates in clinical development [15, 16], highlighting the translational viability of this platform. Collectively, these advances support the potential of a GalNAc‐conjugated siRNA targeting XDH as a promising therapeutic modality for HUA.

Given the unmet need for safe and effective therapies in hyperuricemia (HUA) and the absence of approved siRNA‐based treatments, prior efforts by Alnylam Pharmaceuticals (ALN‐XDH) and Arrowhead Pharmaceuticals (ARO‐XDH) to develop GalNAc‐conjugated small interfering RNAs targeting *XDH* advanced to Phase 1 clinical trials but were ultimately discontinued without public disclosure of detailed efficacy or safety data. These programs nonetheless underscore the therapeutic rationale for inhibiting *XDH*—the key enzyme driving urate overproduction—as a strategy for HUA management. Building on this foundation, we designed YJH‐012‐D, a novel GalNAc‐conjugated siRNA featuring optimized sequence selection and chemical modifications to enhance target specificity and metabolic stability. In the present study, we engineered YJH‐012‐D and systematically evaluated its ability to suppress *XDH* expression, reduce uric acid levels, and maintain a favorable safety profile across primary hepatocytes and preclinical animal models. Comprehensive off‐target assessment was performed using RNA sequencing. Collectively, our data support YJH‐012‐D as a promising next‐generation candidate for the treatment of HUA.

## 2 Experimental Methods and Materials

### 2.1 Cell Culture and Transfection

HepG2 cells (#iCell‐h092; iCell Bioscience Inc., Shanghai, China; RRID: CVCL_0027) were maintained in MEM (#2925213; Gibco, Thermo Fisher Scientific, Waltham, MA, USA) supplemented with 5% FBS (#2556132P; Gibco, Thermo Fisher Scientific) and 1% penicillin–streptomycin (#T321‐100; BDBIO, Shanghai, China). Cells were transfected with 10 nM siRNA using Lipofectamine RNAiMAX (#2515132; Invitrogen, Thermo Fisher Scientific) and harvested 48 h later (three biological replicates per sequence).

Cynomolgus monkey primary hepatocytes (#CCH100Cy‐V10199; Milecell Bio, Shanghai, China) were cultured per the manufacturer's protocol using the provided medium kit. Transfection via spontaneous uptake was initiated 4 h post‐plating (two replicates per concentration), with medium replacement at 48 h and cell collection at 72 h.

### 2.2 Quantitative Real‐Time PCR (qPCR)

Total RNA was extracted from returned cell and tissue samples using a chloroform‐based method and purified with the Total RNA Purification Kit (#BSC69M1E; BIOER, Hangzhou, China). One‐step qPCR was performed using the One‐Step qPCR Kit (#7E0662F4; Vazyme, Nanjing, China). *XDH* mRNA levels were normalized to *GAPDH* and expressed as fold change relative to the model control group.

### 2.3 Western Blot

Liver tissues returned from outsourced studies were homogenized in RIPA lysis buffer containing protease and phosphatase inhibitors (#P1048; Beyotime, Shanghai, China). Total protein was quantified by BCA assay (#20220519; Solarbio, Beijing, China). Equal amounts of protein (20 μg per lane) were separated by SDS‐PAGE on precast gels and transferred to PVDF membranes. Membranes were blocked with 5% nonfat milk and probed with primary antibodies against XDH (#Sc‐398 548; Santa Cruz Biotechnology, Dallas, TX, USA; RRID: AB_3740003) and α‐tubulin (#TA503129; ORIGENE, Rockville, MD, USA; RRID: AB_11127015), followed by HRP‐conjugated secondary antibody (#RS0001; ImmunoWay, Plano, TX, USA; RRID: AB_2943495). Chemiluminescent signals were detected using substrates from Thermo Fisher Scientific.

### 2.4 In Vivo Efficacy Evaluation in Acute Hyperuricemia Mice

All mouse studies used 6–8‐week‐old SPF male C57BL/6 mice (Novopathway) and were approved by the Animal Ethics Committee of Shanghai Paisi Weixin Biotechnology (Approval Nos. P08240118BN2, P08231114AN1, P08231214AN1). Hyperuricemia was induced by daily intraperitoneal injection of potassium oxonate (600 mg/kg; #156124; Sigma, St. Louis, MO, USA), except in vehicle control groups.

In the initial screening study, mice were randomized by body weight into groups (*n* = 10). On Day 1 (D1), animals received a single subcutaneous dose of YJH‐012‐C, ‐D, or ‐E (10 mg/kg; Youjia Biomedical, Hangzhou, China); vehicle and model controls received sterile water. Potassium oxonate was administered daily for 7 or 14 days. On D7 and D14, five mice per group were euthanized 2 h post‐dosing for serum uric acid measurement and liver collection (RNAlater for RNA, snap‐frozen for protein).

In a subsequent dose‐frequency study, mice (*n* = 5/group, 5 groups) received two subcutaneous doses of YJH‐012‐D or vehicle at 3‐day intervals. Hyperuricemia was induced on D7 by a single potassium oxonate injection, and serum uric acid was measured 4 h later.

For long‐term evaluation, mice were assigned to four groups (vehicle control, *n* = 10; others, *n* = 21). From D1 to D42, treatment groups received a single subcutaneous dose of YJH‐012‐D on D1; the positive control received daily oral febuxostat; vehicle and model controls received sterile water. Potassium oxonate was administered daily (except vehicle) starting 30 min post‐dosing. Blood was collected at D4, 7, 10, 14, 21, 28, and 42 (*n* = 3/group) for uric acid, urea, and creatinine; vehicle controls were sampled only on D4 and D42 (*n* = 5). At study termination, serum AST, CK‐MB, and cTnT were assessed, and liver tissues (*n* = 3/group) from vehicle, model, 8 mg/kg YJH‐012‐D, and febuxostat groups underwent H&E staining.

### 2.5 Nonhuman Primate Studies

All cynomolgus monkey studies were conducted under IACUC‐approved protocols at contract research organizations.

For pharmacodynamic assessment, three male monkeys (10–13 years) received a single subcutaneous dose of YJH‐012‐D (10 mg/kg, 0.2 mL/kg) at WuXi AppTec. Hyperuricemia was induced on Days 5 (baseline, prior to dosing), 1, 4, 7, 14, 21, 30, 45, 60, 90, 120, 150, and 180 by coadministration of inosine (100 mg/kg, i.p.) and potassium oxonate (25 mg/kg, s.c.) under conscious conditions. Blood was collected at 1, 2, 4, 6, and 24 h post‐modeling for serum uric acid measurement. Liver biopsies (≥ 5 mg × 2) were obtained at 6 h post‐modeling on Days 7, 21, 60, and 120, as well as pre‐dose, and stored in RNAlater or snap‐frozen at −80°C for sponsor analysis. The study was approved by the IACUC of WuXi AppTec (Approval No. 24020602).

In a separate pharmacokinetic study, 18 cynomolgus monkeys (2–5 years; 3 males and 3 females per group) received single subcutaneous doses of YJH‐012‐D at 3, 10, or 30 mg/kg (1 mL/kg) at Shenzhen InnoStar. Serial blood samples were collected up to 48 h post‐dosing. Liver tissues were harvested from terminal animals at 48, 552, 1056, 1560, 2064, and 2568 h to quantify antisense oligonucleotide concentrations. Plasma and liver levels were determined using a validated LC–MS/MS method, and non‐compartmental pharmacokinetic parameters were calculated with WinNonlin (v8.3). The protocol was approved by the Animal Ethics Committee of Guangxi Fangchenggang Changchun Biotechnology Development Co. Ltd. (Approval No. 240011).

### 2.6 Statistical Analysis

Data are presented as mean ± SD from biological replicates. Group comparisons were performed using unpaired two‐tailed *t*‐tests (GraphPad Prism 8, San Diego, CA, USA). A *p* value < 0.05 was considered statistically significant. Inhibition rates for both XDH mRNA and protein were calculated as (1 − relative expression) × 100%.

## 3 Result

### 3.1 Optimization and In Vitro Screening of Chemically Modified siRNAs Targeting XDH

**FIGURE 1:**
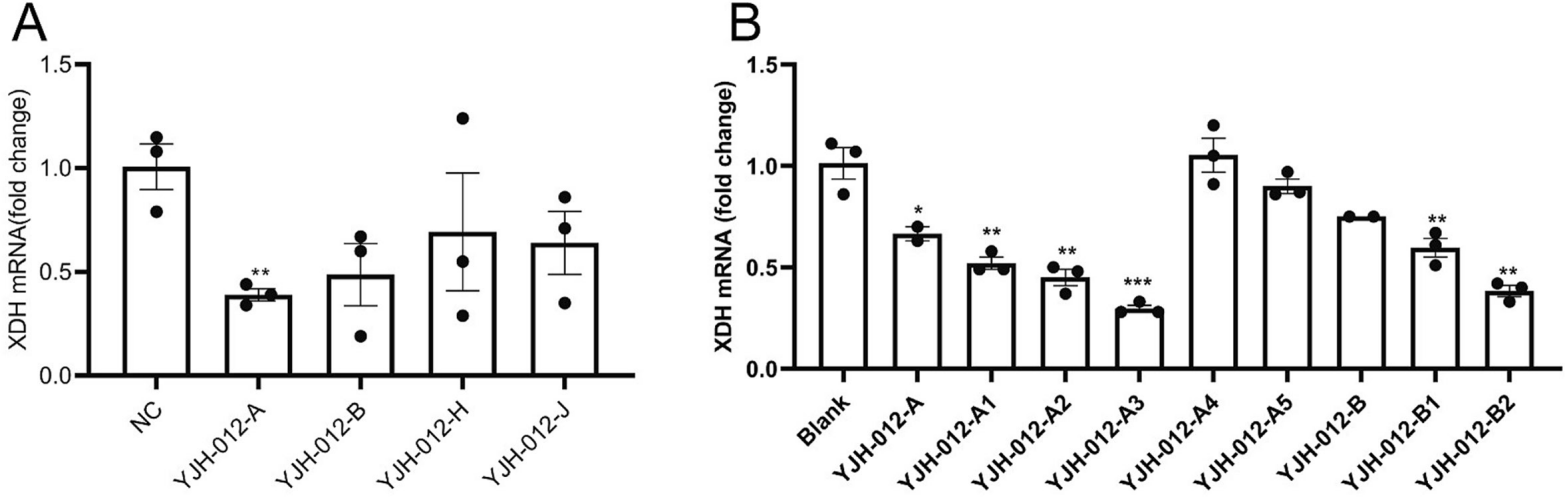
Silencing efficiency of unmodified and chemically modified *XDH* siRNAs in HepG2 Cells. (A) Silencing efficiency of unmodified *XDH* siRNAs (10 nM) transfected into HepG2 cells for 48 h, evaluated by qPCR (*n* = 3; one‐tailed *t*‐test vs. NC group). (B) Silencing efficiency of modified *XDH* siRNAs (10 nM) transfected into HepG2 cells for 48 h, evaluated by qPCR (*n* = 3; one‐tailed *t*‐test vs. blank group). *p < 0.05, **p < 0.01, ****p < 0.0001; one‐way ANOVA. Data are mean ± SD.

Four pairs of small interfering RNAs (siRNAs) from the YJH‐012 series, each targeting a distinct region of human *XDH* mRNA, were designed and synthesized. Transfection into HepG2 cells at 10 nM using Lipofectamine RNAiMAX followed by qPCR analysis at 48 h post‐transfection revealed that YJH‐012‐A and YJH‐012‐B achieved the greatest suppression of *XDH* mRNA, reducing expression by 61.00% and 51.33%, respectively (Figure 1A). To enhance nuclease resistance and reduce immunostimulatory potential, these two lead sequences were chemically modified (Figure S1) [17], yielding nine derivative siRNAs. A secondary screen in HepG2 cells at the same concentration identified YJH‐012‐A3, YJH‐012‐B2, and YJH‐012‐A2 as the most potent variants, with *XDH* mRNA inhibition rates of 70.33%, 61.67%, and 55.00%, respectively, after 48 h (Figure 1B).

### 3.2 Potency and Dose–Response Profile of Lead *XDH*‐siRNAs in Primary Hepatocytes

**FIGURE 2:**
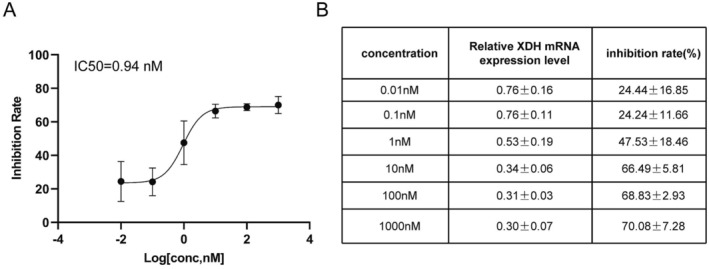
Validation of knockdown efficiency in primary hepatocytes. (A) IC_50_ curve of YJH‐012‐D in cynomolgus monkey primary hepatocytes 72 h post‐transfection at varying concentrations (*n* = 2, mean ± SD). (B) Relative XDH mRNA expression levels and inhibition rates at various concentrations.

Given that hepatic xanthine dehydrogenase (XDH) is the primary enzymatic source of urate overproduction in hyperuricemia [18], the three most potent siRNA candidates identified in HepG2 cells—YJH‐012‐A2, YJH‐012‐A3, and YJH‐012‐B2—were conjugated to N‐acetylgalactosamine (GalNAc) using established methods to facilitate hepatocyte uptake, yielding YJH‐012‐C, D, and E, respectively. Among these, YJH‐012‐D was selected for dose–response evaluation in primary cynomolgus monkey hepatocytes. Following passive uptake across a concentration range of 0.01–1000 nM, *XDH* mRNA inhibition increased in a dose‐dependent manner, reaching 24.44%, 24.24%, 47.53%, 66.49%, 68.83%, and 70.08% at 72 h, respectively (Figure 2B). The calculated IC_50_ for *XDH* mRNA suppression was 0.94 nM (Figure 2A).

### 3.3 Evaluation of Sequence‐Dependent Off‐Target Risk for YJH‐012‐A3 Using RNA‐Seq and BLAST Analysis

**FIGURE 3:**
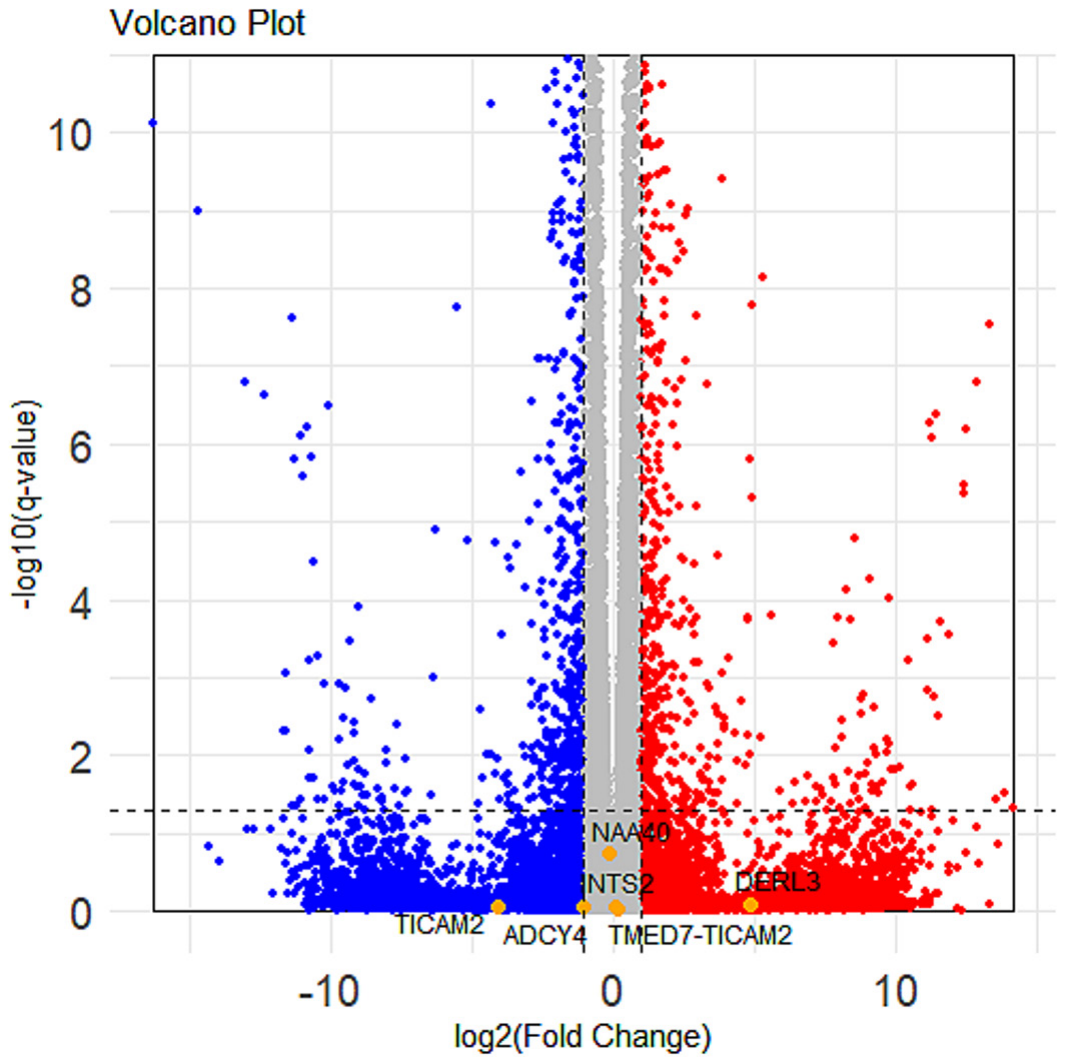
Off‐target analysis and RNA‐seq of YJH‐012‐A3. The sequencing results of YJH‐012‐A3.

To evaluate the potential for sequence‐dependent, miRNA‐like off‐target effects, transcriptome‐wide RNA sequencing (RNA‐seq) was performed on HepG2 cells treated with YJH‐012‐A3, and the results were integrated with in silico off‐target predictions. Differentially expressed genes were filtered to include only protein‐coding transcripts showing ≥ 50% downregulation with a *p* value ≤ 0.05. Separately, potential off‐target genes were predicted by BLAST alignment of both the sense and antisense strands of YJH‐012‐A3 against the human RefSeq database (NCBI). Cross‐comparison of the RNA‐seq–derived gene list with the BLAST‐predicted off‐targets revealed no overlapping genes, indicating a low risk of sequence‐specific, miRNA‐like silencing (Figure 3).

### 3.4 In Vivo Identification and Dose Optimization of YJH‐012‐D, a Potent *XDH*‐Targeting siRNA, in Hyperuricemic Mice

**FIGURE 4:**
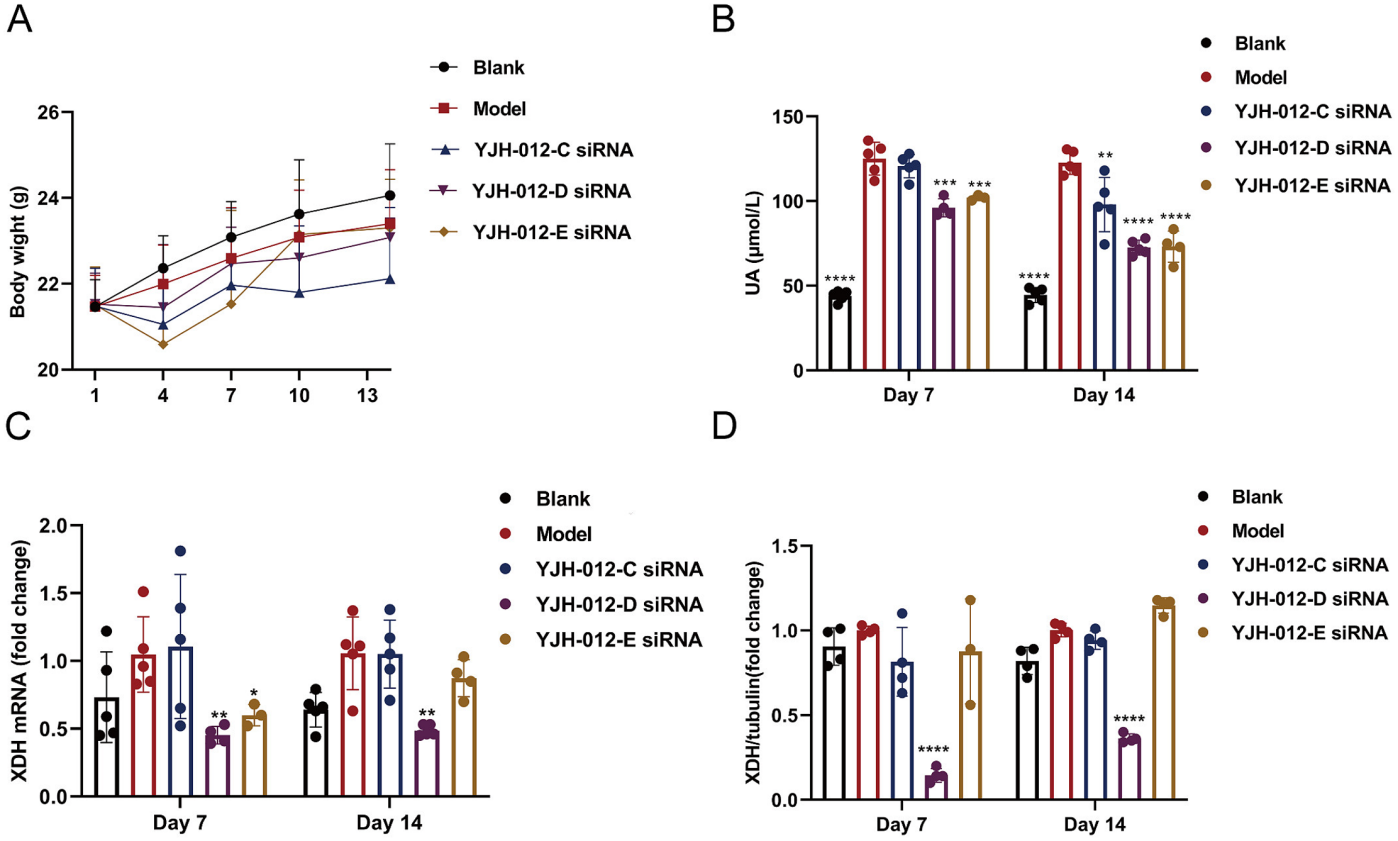
In vivo efficacy of GalNAc‐siRNA targeting XDH in hyperuricemic mice. (A) Body weight changes in C57BL/6 mice (*n* = 10/group on Day 1 and 4; Day 7: *n* = 10 for blank, model and YJH‐012‐C groups, *n* = 9 for YJH‐012‐D; *n* = 7 for YJH‐012‐E; Days 10 and 14: *n* = 5 for all groups except YJH‐012‐E [*n* = 4]). (B) Changes in serum uric acid levels in mice on Day 7 and 14 after a single subcutaneous injection (Day 7 and 14: *n* = 5 for all groups except YJH‐012‐D [*n* = 4 on Day 7], YJH‐012‐E [*n* = 3 on Day 7; *n* = 4 on Day 14]). (C) Relative XDH mRNA expression levels in liver tissue on D7 and D14 after a single administration of YJH‐012‐modified siRNA (Day 7 and 14: *n* = 5 for all groups except YJH‐012‐D [*n* = 4 on Day 7], YJH‐012‐E [*n* = 3 on Day 7; *n* = 4 on Day 14]). (D) Relative XDH protein expression levels in liver tissue on D7 and D14 after a single administration of YJH‐012‐modified siRNA (Day 7: *n* = 4 for all groups except YJH‐012‐E [*n* = 3]; Day 14: *n* = 4 for all groups). *p < 0.05, **p < 0.01, ***p < 0.001, ****p < 0.0001 versus Model group; one‐way ANOVA. Data are mean ± SD.

Based on the potent *XDH* knockdown observed in vitro, a potassium oxonate–induced hyperuricemic mouse model was used to evaluate the in vivo efficacy of YJH‐012‐C, YJH‐012‐D, and YJH‐012‐E. Mice received a single subcutaneous injection of each siRNA at 8 mg/kg, and serum uric acid (UA) levels were measured on Days 7 and 14, with hepatic *XDH* mRNA and protein expression assessed concurrently. Throughout the study, mice in both the blank control and model control groups remained in good health and exhibited steady weight gain (Figure 4A). As expected, the model control group showed a significant elevation in serum UA compared with the blank control (*p* < 0.0001), confirming successful induction of hyperuricemia (Figure 4B). By Day 14, all three siRNA‐treated groups demonstrated significant reductions in serum UA relative to the model control, with decreases of 20.15% (YJH‐012‐C), 40.82% (YJH‐012‐D), and 40.35% (YJH‐012‐E) (Figure 4B). Consistent with qPCR and Western blot analyses (Figure 4C,D), YJH‐012‐D exhibited the most robust and sustained suppression of both hepatic *XDH* mRNA and protein. It was therefore selected for further dose optimization.

**FIGURE 5:**
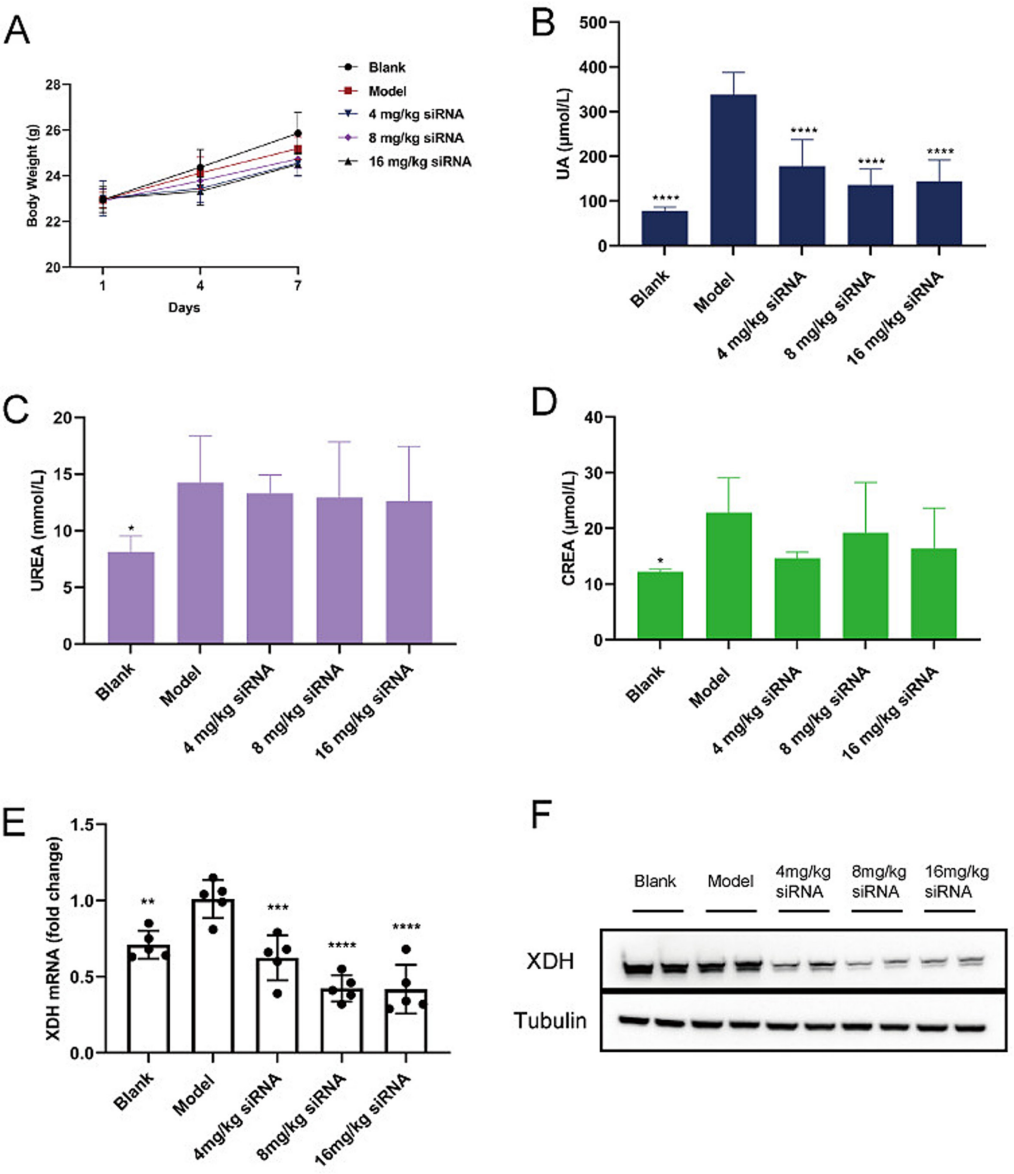
In vivo efficacy evaluation of YJH‐012‐D siRNA in mice. Serum was collected for UA, UREA, and CREA measurement at 4 h post‐modeling from each group. (A) Changes in body weight (*n* = 5). (B) Changes in serum uric acid (UA) levels (*n* = 5). (C) Changes in blood urea nitrogen (UREA) levels (*n* = 5). (D) Changes in serum creatinine (CREA) levels (*n* = 5). (E) Relative XDH mRNA expression levels in liver tissue 7 days post‐administration of YJH‐012‐D (*n* = 5). (F) The protein expression levels in liver tissue 7 days post‐administration of YJH‐012‐D were determined by western blot. *p < 0.05, **p < 0.01, ***p < 0.001, ****p versus Model group; one‐way ANOVA. Data are mean ± SD.

To determine the optimal therapeutic dose of YJH‐012‐D, mice with potassium oxonate–induced acute hyperuricemia were administered a single subcutaneous injection at 4, 8, or 16 mg/kg and evaluated 7 days post‐dosing. All groups showed weight gain, though the model control and treated groups gained slightly less than the blank control (no statistically significant differences; Figure 5A). Serum UA levels were significantly reduced by all doses compared with the model group, with no additional benefit observed at 16 mg/kg versus 8 mg/kg (Figure 5B). Renal function markers (serum creatinine and urea) remained unchanged across groups (Figure 5C,D), indicating no overt toxicity. Hepatic *XDH* mRNA and protein expression were markedly suppressed at all tested doses, with 8 and 16 mg/kg showing comparable and superior efficacy to 4 mg/kg (Figure 5E,F). Collectively, these results demonstrate that a single 8 mg/kg dose of YJH‐012‐D achieves maximal *XDH* suppression and urate‐lowering efficacy in hyperuricemic mice.

### 3.5 Sustained Urate‐Lowering Effect and Safety of a Single Dose of YJH‐012‐D in a Chronic Hyperuricemia Mouse Model

**FIGURE 6:**
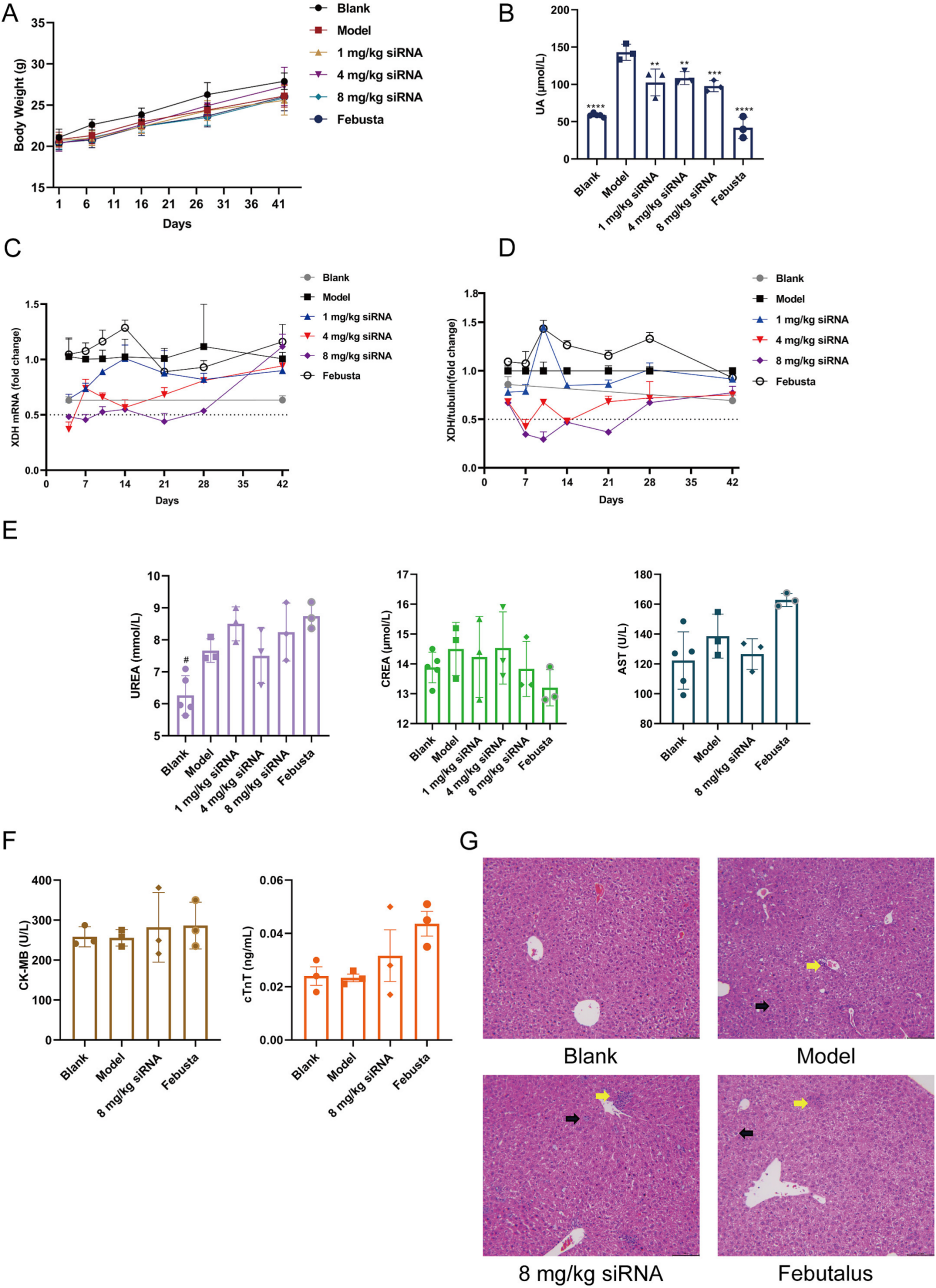
Long‐term efficacy and safety of YJH‐012‐D in a chronic hyperuricemia mouse model. (A) Changes in body weight (Blank group: *n* = 10 on Day 1, *n* = 5 on other days. Other groups: *n* = 21 on Day 1, *n* = 18 on Day 7, *n* = 9 on Day 16, *n* = 28 on Day 28, *n* = 3 on Day 42). (B) Changes in serum uric acid (UA) levels at 42 days posttreatment (Blank group: *n* = 5; other groups: *n* = 3). (C) Relative XDH mRNA expression levels in liver tissue (Blank group: *n* = 5; other groups: *n* = 3). (D) Relative XDH protein expression levels in liver tissue (Blank group: *n* = 2 on Day 4, *n* = 5 on Day 42. Other groups: *n* = 2 on Days 4, 7, 10, 14, 21 [except 1 mg/kg group, *n* = 1 on Day 10]; *n* = 3 on Days 28 and 42). (E) Changes in serum levels of UREA, CREA, AST at 42 days posttreatment (Blank group: *n* = 5; other groups: *n* = 3). (F) Changes in serum levels of CK‐MB, cTnT at 42 days posttreatment (*n* = 3). (G) Hematoxylin and eosin (H&E) staining of mouse liver tissue (100× magnification; yellow arrows indicate inflammatory cell infiltration; black arrows indicate cellular edema). *p < 0.05, **p < 0.01, ***p < 0.001, ****p versus Model group; one‐way ANOVA. Data are mean ± SD.

To assess the duration of action and safety of YJH‐012‐D, a chronic hyperuricemia model was established in mice by daily intraperitoneal injection of potassium oxonate (600 mg/kg) for 42 consecutive days. All groups except the blank control received this treatment. Mice were administered a single subcutaneous dose of YJH‐012‐D at 1, 4, or 8 mg/kg on Day 0, while the positive control group received oral febuxostat once daily throughout the study period. Throughout the experiment, all animals remained in good health and exhibited steady weight gain (Figure 6A), and sustained hyperuricemia was confirmed in model groups (Figure 6B).

All YJH‐012‐D dose groups significantly reduced serum uric acid levels compared with the model control (Figure 6B). To evaluate silencing kinetics, liver tissues were collected on Days 4, 7, 10, 14, 21, 28, and 42 from three randomly selected mice per group. qPCR and Western blot analyses revealed dose‐dependent suppression of hepatic *XDH* mRNA and protein that persisted for the full 42‐day duration (Figure 6C,D).

Comprehensive safety assessment—including serum markers of renal (UREA, CREA) and cardiac (AST, CK‐MB, cTnT) function, as well as histopathological examination of heart and kidney (H&E staining)—showed no significant abnormalities (Figure 6E–G). Collectively, these results demonstrate that a single subcutaneous administration of YJH‐012‐D achieves potent and sustained urate‐lowering effects over 6 weeks with a favorable safety profile.

### 3.6 Sustained Urate‐Lowering Efficacy and Favorable Hepatic Pharmacokinetics of YJH‐012‐D in Cynomolgus Monkeys

**FIGURE 7:**
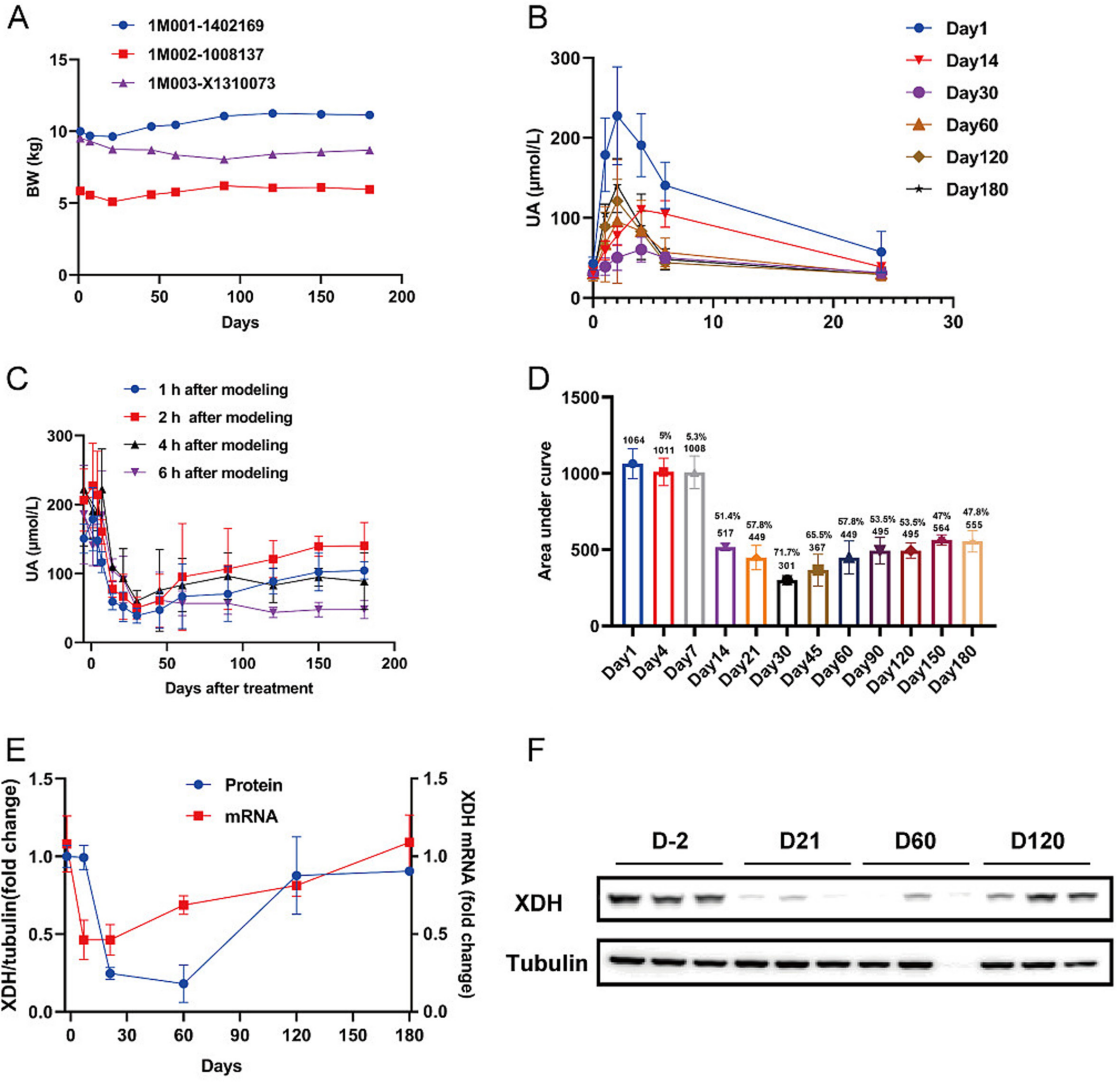
Efficacy of YJH‐012‐D in cynomolgus monkeys following subcutaneous administration. (A) Changes in animal body weight (kg). Weights were recorded once before dosing, once before each modeling event, and weekly otherwise. (B) Serum uric acid (UA) levels at various time points post‐dose, measured before modeling and at 1, 2, 4, 6, and 24 h post‐modeling (*n* = 3, mean ± SEM). (C) Serum uric acid (UA) levels at 1, 2, 4, and 6 h post‐modeling across different time points post‐dose (*n* = 3, mean ± SEM). (D) Area under the curve (AUC) for serum uric acid (UA) levels from pre‐dose to 6 h post‐modeling (AUCpre‐6 h) at different time points post‐dose (*n* = 3, mean ± SEM). Percentages indicate uric acid (UA) inhibition rates calculated relative to the Day 1 baseline. (E) Relative XDH mRNA and protein expression levels in liver tissue at various time points after a single administration of YJH‐012‐D (*n* = 3 per time point for both assays, except for the protein assay at Day 60, where *n* = 2; mean ± SD). (F) The XDH protein expression levels in liver tissue at various time points after a single administration of YJH‐012‐D.

To evaluate the translational potential of YJH‐012‐D, a single subcutaneous dose (10 mg/kg) was administered to three male cynomolgus monkeys. Following an uric acid–elevating challenge (method described in Section 2), serum uric acid (UA) levels were markedly reduced, with maximal suppression (~74%–76%) observed at 1–6 h post‐challenge on Day 30 (Figure 7C). Notably, UA levels remained below baseline throughout the 180‐day observation period (Figure 7D), demonstrating durable pharmacodynamic activity. Liver biopsies revealed sustained suppression of both *XDH* mRNA and protein for at least 120 days (Figure 7E,F), consistent with the prolonged urate‐lowering effect.

Pharmacokinetic analysis following single subcutaneous doses of 3, 10, and 30 mg/kg in separate cohorts showed rapid plasma clearance (Cl__F_ob_: 1100 → 428 mL/h/kg with increasing dose) and high hepatic accumulation. Liver *C*_max_ values reached 17 700, 50 600, and 131 000 ng/mL, respectively, yielding liver‐to‐plasma AUC_last_ ratios of 2000–6100 (Table 1). The estimated hepatic half‐life exceeded 10 days—substantially longer than in plasma—supporting the observed duration of efficacy. These data indicate that YJH‐012‐D achieves preferential liver exposure and prolonged target engagement, underpinning its sustained therapeutic effect.

**TABLE 1:** Summary table of AS pharmacokinetic parameters.

Group	Plasma						Hepatic					
	1		2		3		1		2		3	
Dose (mg/kg)	3		10		30		3		10		30	
PK parameters	Mean ± SD	*N*	Mean ± SD	*N*	Mean ± SD	*N*	Mean ± SD	*N*	Mean ± SD	*N*	Mean ± SD	*N*
AUC_INF_obs_ (h*ng/mL)	1240 ± 314	5	6110 ± 622	5	31 300 ± 6250	6	7 660 000 ± 1 730 000	3	21 100 000 ± 2 590 000	6	62 900 000 ± 32 200 000	6
AUC_last_ (h*ng/mL)	1100 ± 281	6	5350 ± 1010	6	30 700 ± 6000	6	6 690 000 ± 1 090 000	6	21 000 000 ± 2 590 000	6	61 400 000 ± 29 100 000	6
*C*_max_ (ng/mL)	269 ± 62.6	6	1060 ± 298	6	3920 ± 1650	6	17 700 ± 3310	6	50 600 ± 6770	6	131 000 ± 25 600	6
Cl__F_ob_ (mL/h/kg)	1100 ± 251	5	711 ± 81.3	5	428 ± 82.6	6	—	—	—	—	—	—
*T*_1/2_ (h)	2.25 ± 0.806	4	2.19 ± 0.641	4	3.17 ± 0.530	6	NC	1	269 ± 37.9	6	315 ± 178	6
*T*_max_ (h)	0.750 (0.500, 2.00)	6	1.00 (0.500, 2.00)	6	1.00 (0.500, 2.00)	6	48.0 (48.0, 48.0)	6	48.0 (48.0, 48.0)	6	48.0 (48.0, 48.0)	6

*Note:* NC—More than 50% of the parameters are unavailable and not included in the calculation. *T*_max_ (h)—median (min, max).

## 4 Discussion

The development of RNA interference (RNAi)–based therapeutics has opened new avenues for the treatment of metabolic disorders, including hyperuricemia (HUA). In this study, we report the preclinical characterization of YJH‐012‐D, a N‐acetylgalactosamine (GalNAc)–conjugated small interfering RNA (siRNA) targeting hepatic xanthine dehydrogenase (*XDH*), a key enzyme in urate biosynthesis. Our findings demonstrate that YJH‐012‐D induces potent, durable, and liver‐specific suppression of *XDH*, resulting in sustained reductions in serum uric acid levels in both murine and nonhuman primate models.

In a potassium oxonate–induced chronic hyperuricemia mouse model, a single subcutaneous dose of YJH‐012‐D (8 mg/kg) significantly lowered serum uric acid and suppressed hepatic *XDH* mRNA and protein expression for over 42 days (Figure 6C,D). This prolonged pharmacodynamic effect aligns with its favorable pharmacokinetic profile, which exhibited a liver‐to‐plasma area under the curve (AUC) ratio ranging from 2000 to 6100 and an estimated hepatic half‐life exceeding 10 days (Table 1). These data support the hypothesis that GalNAc‐mediated hepatocyte targeting enables efficient intracellular delivery of siRNA, facilitating sustained gene silencing with minimal systemic exposure.

In cynomolgus monkeys, a single 10 mg/kg subcutaneous dose of YJH‐012‐D produced a uric acid–lowering effect that persisted for up to 180 days (Figure 7D), with *XDH* inhibition maintained for at least 120 days (Figure 7E,F). The extended duration of action underscores the potential for infrequent dosing in clinical practice—a feature that may enhance patient adherence and long‐term therapeutic outcomes. Importantly, comprehensive safety evaluations—including clinical biochemistry, histopathological examination, and transcriptomic profiling—revealed no evidence of significant off‐target effects or organ toxicity, highlighting the specificity and favorable safety profile of this RNAi‐based approach.

Compared with conventional xanthine oxidase inhibitors such as febuxostat and allopurinol, YJH‐012‐D offers several potential advantages. First, it acts at the mRNA level to suppress *XDH* expression, thereby reducing urate production more comprehensively than reversible enzymatic inhibition. Second, GalNAc‐conjugation ensures preferential hepatic delivery, minimizing systemic exposure and off‐target pharmacological effects, which may improve the therapeutic index. Third, the sustained duration of action supports dosing intervals far less frequent than daily oral regimens, a considerable benefit in the management of chronic conditions like HUA.

Despite these promising results, certain limitations warrant acknowledgment. The present study was conducted in preclinical models that do not fully recapitulate the long‐term disease progression or common comorbidities (e.g., hypertension, renal impairment) associated with human hyperuricemia. Moreover, the exploratory monkey study lacked a concurrent control group, limiting definitive attribution of observed physiological changes—such as transient weight fluctuations—to YJH‐012‐D treatment. Future investigations in more pathophysiologically relevant models, along with controlled clinical trials, will be essential to validate the translational potential of this candidate.

In conclusion, YJH‐012‐D represents a novel, potent, and well‐tolerated RNAi therapeutic candidate for hyperuricemia. Its liver‐targeted, long‐acting mechanism of action provides a compelling rationale for its continued clinical development as a next‐generation therapy for patients with HUA.

## Author Contributions

**Tingke Tang:** methodology, writing – review and editing, writing – original draft. **Qiuying Li:** writing – original draft, validation, writing – review and editing, visualization. **Chenguang Zhang:** investigation, writing – review and editing, data curation, visualization. **Yongxing Wang:** conceptualization, investigation, methodology. **Lu Yang:** methodology, formal analysis, data curation. **Haibin Huang:** investigation, formal analysis, conceptualization. **Yujun He:** methodology, validation, formal analysis. **Zhi Zheng:** data curation, investigation, validation. **Dong Liang:** writing – review and editing, methodology, investigation. **Meng Wang:** project administration, writing – review and editing, supervision. **Yingyu Wu:** investigation, methodology, formal analysis, supervision. **Chengjiang Zhao:** writing – review and editing, investigation, project administration, validation. **Wenhao Cui:** project administration, writing – review and editing, supervision, conceptualization.

## Conflicts of Interest

The authors declare no conflicts of interest.

## Supporting information


**Figure S1:** Modification patterns of siRNA.

## Data Availability

The data that support the findings of this study are available from the corresponding author upon reasonable request.

## References

[1] R. Wang , M. Halimulati , X. Huang , Y. Ma , L. Li , and Z. Zhang , “Sulforaphane‐Driven Reprogramming of Gut Microbiome and Metabolome Ameliorates the Progression of Hyperuricemia,” Journal of Advanced Research 52 (2023): 19–28. 36371056 10.1016/j.jare.2022.11.003 PMC10555773

[2] L. Du , Y. Zong , H. Li , et al., “Hyperuricemia and Its Related Diseases: Mechanisms and Advances in Therapy,” Signal Transduction and Targeted Therapy 9, no. 1 (2024): 212. 39191722 10.1038/s41392-024-01916-y PMC11350024

[3] R. Terkeltaub , “Gout. Novel Therapies for Treatment of Gout and Hyperuricemia,” Arthritis Research & Therapy 11, no. 4 (2009): 236. 19664185 10.1186/ar2738 PMC2745774

[4] Y. Li , Z. Shen , B. Zhu , H. Zhang , X. Zhang , and X. Ding , “Demographic, Regional and Temporal Trends of Hyperuricemia Epidemics in Mainland China From 2000 to 2019: A Systematic Review and Meta‐Analysis,” Global Health Action 14, no. 1 (2021): 1874652. 33475474 10.1080/16549716.2021.1874652 PMC7833047

[5] Y. Wang , S. Liu , S. Tian , et al., “C1QBP Regulates Apoptosis of Renal Cell Carcinoma via Modulating Xanthine Dehydrogenase (XDH) Mediated ROS Generation,” International Journal of Medical Sciences 19, no. 5 (2022): 842–857. 35693733 10.7150/ijms.71703 PMC9149634

[6] T. Nishino , “XDH and XO Research and Drug Discovery‐Personal History,” Molecules 28, no. 11 (2023): 4440. 37298917 10.3390/molecules28114440 PMC10254830

[7] C. Wu , A. R. Wong , Q. Chen , et al., “Identification of Inhibitors From a Functional Food‐Based Plant Perillae Folium Against Hyperuricemia via Metabolomics Profiling, Network Pharmacology and All‐Atom Molecular Dynamics Simulations,” Frontiers in Endocrinology 15 (2024): 1320092. 38435751 10.3389/fendo.2024.1320092 PMC10905266

[8] Y. Afinogenova , A. Danve , and T. Neogi , “Update on Gout Management: What Is Old and What Is New,” Current Opinion in Rheumatology 34, no. 2 (2022): 118–124. 34907116 10.1097/BOR.0000000000000861 PMC8799507

[9] W. B. White , K. G. Saag , M. A. Becker , et al., “Cardiovascular Safety of Febuxostat or Allopurinol in Patients With Gout,” New England Journal of Medicine 378, no. 13 (2018): 1200–1210. 29527974 10.1056/NEJMoa1710895

[10] J. K. Nair , H. Attarwala , A. Sehgal , et al., “Impact of Enhanced Metabolic Stability on Pharmacokinetics and Pharmacodynamics of GalNAc‐siRNA Conjugates,” Nucleic Acids Research 45, no. 19 (2017): 10969–10977. 28981809 10.1093/nar/gkx818 PMC5737438

[11] D. B. Harmon , W. K. Mandler , I. J. Sipula , et al., “Hepatocyte‐Specific Ablation or Whole‐Body Inhibition of Xanthine Oxidoreductase in Mice Corrects Obesity‐Induced Systemic Hyperuricemia Without Improving Metabolic Abnormalities,” Diabetes 68, no. 6 (2019): 1221–1229. 30936145 10.2337/db18-1198 PMC6610025

[12] M. Friedrich and A. Aigner , “Therapeutic siRNA: State‐of‐the‐Art and Future Perspectives,” BioDrugs 36, no. 5 (2022): 549–571. 35997897 10.1007/s40259-022-00549-3 PMC9396607

[13] A. D. Springer and S. F. Dowdy , “GalNAc‐siRNA Conjugates: Leading the Way for Delivery of RNAi Therapeutics,” Nucleic Acid Therapeutics 28, no. 3 (2018): 109–118. 29792572 10.1089/nat.2018.0736 PMC5994659

[14] B. Hu , L. Zhong , Y. Weng , et al., “Therapeutic siRNA: State of the Art,” Signal Transduction and Targeted Therapy 5, no. 1 (2020): 101. 32561705 10.1038/s41392-020-0207-x PMC7305320

[15] J. F. Alterman , K. Y. Gross , and A. Khvorova , “The Approval of Redemplo for Familial Chylomicronemia Syndrome and the Many Flavors of GalNAc‐Oligonucleotides,” Nucleic Acid Therapeutics 36, no. 3 (2026): 129–134. 41877501 10.1177/21593337261435980

[16] Q. V. Le and G. Shim , “Evolution of siRNA Therapeutics: From Mechanistic Foundations to Clinical Expansion,” Pharmaceutics 18, no. 5 (2026): 593. 42198287 10.3390/pharmaceutics18050593 PMC13210848

[17] Y. Dong , D. J. Siegwart , and D. G. Anderson , “Strategies, Design, and Chemistry in siRNA Delivery Systems,” Advanced Drug Delivery Reviews 144 (2019): 133–147. 31102606 10.1016/j.addr.2019.05.004 PMC6745264

[18] N. Liang , X. Yuan , L. Zhang , et al., “Fatty Acid Oxidation‐Induced HIF‐1alpha Activation Facilitates Hepatic Urate Synthesis Through Upregulating NT5C2 and XDH,” Life Metabolism 3, no. 5 (2024): loae018. 39872146 10.1093/lifemeta/loae018 PMC11749550

