# Supplementary figures and images for: Potent and Sustained Lowering of Serum Uric Acid by YJH‐012‐D, a GalNAc‐Conjugated siRNA Targeting Xanthine Dehydrogenase
Source: Pharmacol Res Perspect. 2026 Jul 31;14(4):e70308. doi: 10.1002/prp2.70308 (PMC13428038; doi:10.1002/prp2.70308)

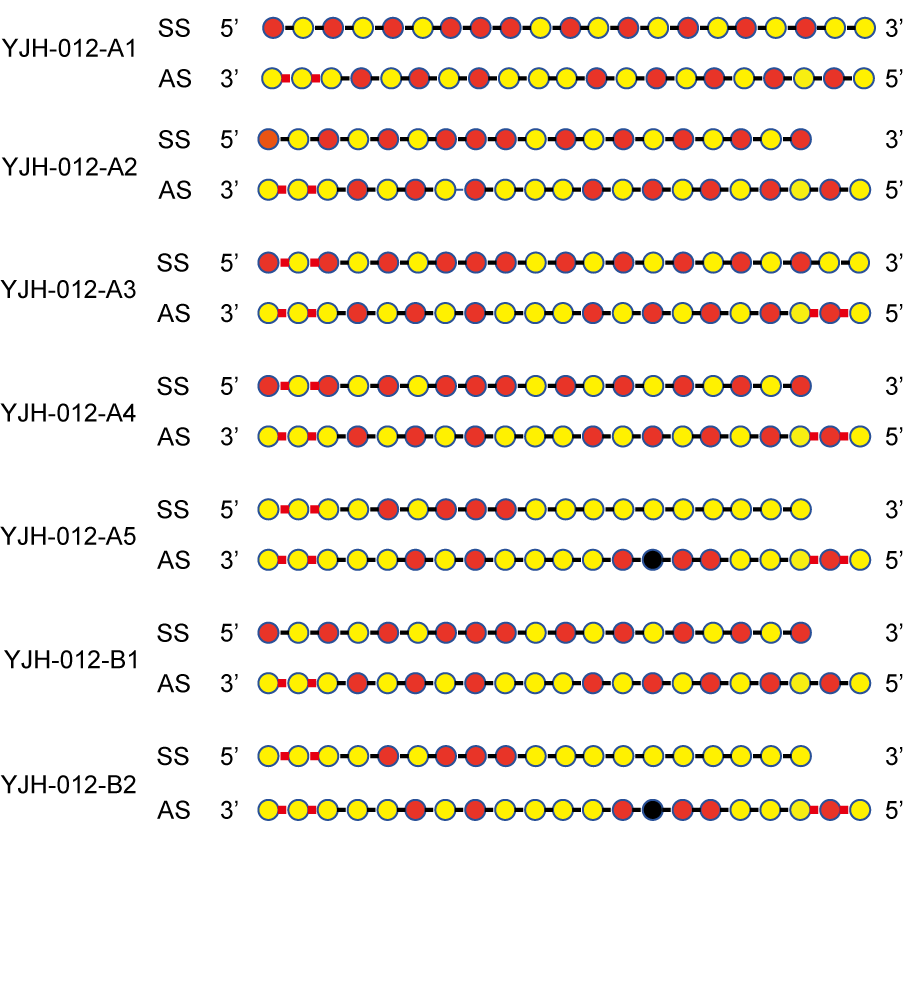


Figure S1. Modification patterns of siRNA.

2'-F

phosphorothio

2'-OME

GNA

Supplement: Supplementary file 1 — Figure S1: Modification patterns of siRNA. [file PRP2-14-e70308-s001.docx]
